# Inflammatory markers in end-stage renal disease patients on haemodialysis

**DOI:** 10.5937/jomb0-25120

**Published:** 2020-10-02

**Authors:** Phebe Lotfy Abdel-Messeih, Manal Mohamed Alkady, Neveen Mostafa Nosseir, Mohamed Said Tawfik

**Affiliations:** 1 Egyptian Atomic Energy Authority (EAEA), National Centers for Radiation Research and Technology (NCRRT), Health Radiation Research Department, Clinical Pathology Unit, Egypt; 2 Egyptian Atomic Energy Authority (EAEA), National Centers for Radiation Research and Technology (NCRRT), Health Radiation Research Department, Internal Medicine Unit, Cairo, Egypt

**Keywords:** CRP, ESRD, CXCL16, iPTH, iPTH, CXCL16, ESRD, CRP

## Abstract

**Background:**

CXC chemokine ligand 16 (CXCL16) is an inflammatory chemokine that mediates renal infiltration of macrophages and activated T cells. Aim: To investigate serum levels of CXCL16 in patients undergoing hemodialysis and their correlation with other inflammatory markers such as C-reactive protein (CRP) and intact parathyroid hormone (iPTH).

**Methods:**

The study included 40 hemodialysis patients (22 males) and 40 age and gender-matched controls (24 males). Fasting blood sugar (FBS), urea, creatinine, calcium and inorganic phosphorous were assayed in participants using routine methods, glycosylated hemoglobin (HbA1c) by quantitative chromatographic spectrophotometry, iPTH by chemiluminescent microparticle immunoassay, CRP by nephelometry and CXCL16 by ELISA technique.

**Results:**

Serum CXCL16, CRP, PTH, FBS, HbA1c, phosphorus, urea, and creatinine levels were significantly higher in hemodialysis patients compared to controls (p<0.00001). No statistically significant differences were observed between patients and controls for calcium. Serum CXCL16 levels correlated positively with CRP (r=0.956, p<0.00001) and iPTH (r=-0.403, p<0.001). Hemodialysis patients (diabetics or hypertensives) had significantly higher CXCL16 levels compared to non-diabetics or non-hypertensives.

**Conclusions:**

High levels of serum CXCL16, CRP and iPTH reflect the inflammatory status of hemodialysis patients and help avoid complications. Serum CXCL16 could be used as a biomarker together with CRP in these patients.

## Abstract (sr)

**Uvod:**

CXC hemokin ligand 16 (CXCL16) je infalamatorni hemokin koji posreduje u bubrežnoj infiltraciji makrofaga i aktivira T ćelije. Cilj: Svrha ovog rada je bila da se ispitaju nivoi serumskog CXCL16 u pacijenata koji su bili na hemodijalizi i njihova korelacija sa drugim inflamatornim markerima kao što su C-reaktivni protein (CRP) i intaktni paratireoidni hormon (iPTH).

**Metode:**

Izučavanje je obuhvatilo 40 pacijenata na hemodijalizi (22 muškarca) i kontrolnu grupu starosti 40 godina (24 muškarca). Glukoza na tašte (FBS), ureja, kreatinin, kalcijum i neorganski fosfat su kod ispitanika određivani rutinskim metodama, glikozilirani hemoglobin (HbA1c) primenom kvantitativne hromatografske spektrometrije, iPTH hemiluminiscentnim imunoodređivanjem, CRP nefelometrijski i CXCL16 primenom ELISA tehnike.

**Rezultati:**

Nivoi CXCL16, CRP, iPTH, FBS, ureje i kreatinina su bili značajno viši kod pacijenata na hemodijalizi u poređenju sa kontrolnom grupom (p < 0,00001). Nisu nađene statistički značajne razlike između pacijenata i kontrolne grupe za vrednosti kalcijuma, fosfora i HbA1c. Nivoi CXC16 bili su u pozitivnoj korelaciji sa vrednostima CRP (r = 0,956, p < 0,00001) i iPTH (r = - 0,403, p < 0,001). Pacijenti na hemodijalizi (dijabetičari ili hipertenzivni) imali su značajno više nivoe u poređenju sa ne-dijabetičarima ili sa pacijentima koji nisu bili hipertenzivni.

**Zaključak:**

Visoki nivoi serumskog CXCL16, CRP i iPTH odražavaju inflamatorni status pacijenata na hemodijalizi i pomažu izbegavanju komplikacija. Serumski CXCL16 može da se koristi kao biomarker zajedno sa CRP kod ovih pacijenata.

## Introduction

Chronic kidney disease (CKD) is recognized as a syndrome that carries a high risk of morbidity and mortality. The consequent kidney tissue damage may end up with the patient depending on lifelong hemodialysis [1]. The chief causes of CKD are diabetes mellitus and hypertension, which are involved in up to two-thirds of cases [2]. The progression to endstage renal disease (ESRD) is defined by an estimated glomerular filtration rate (eGFR) less than 15 mL/min per 1.73 m 2 and undergoing renal replacement therapy or dialysis treatment for survival [2]. Chronic kidney disease is the principal and one of the quickest growing causes of mortality all over the globe [3].

Cardiovascular disease (CVD) is on the list of the leading causes of mortality in CKD patients [4]. Nevertheless, the exact etiology linking CKD with CVD remains poorly understood and consequently, therapy nowadays is regarded as being unsatisfactory. A chronic low-grade systemic inflammation in addition to dyslipidemias are believed to play a major role [5].

Chemokine ligand-16 (CXCL16) is a small cytokine categorized as being a part of the CXC chemokine family. It combines scavenger receptor functions with properties of an inflammatory chemokine [5] [6]. It exists in a transmembrane as well as a soluble form. The transmembrane form is comprised of a chemokine domain, a mucin-like stalk, a transmembrane domain, as well as a cytoplasmic tail. The soluble form results from cleavage at the cell surface and is composed of an extracellular stalk and chemokine domain [7]. The transmembrane form functions as an adhesion molecule for CXCL16 expressing cells and as a scavenger receptor for pathogenic oxidized low-density lipoproteins (oxLDL). The soluble form is regarded as a chemoattractant that enhances the migration of CXC chemokine receptor type 6 (CXCR6) expressing cells including T cells, monocytes, and myeloid fibroblasts [8]. Trans membrane CXCL16 has been found to be present on glomerular and tubular cells during renal injury [9].

Approximately 30 to 50% of CKD cases have been found to have noticeably raised levels of serum inflammatory biomarkers including C-reactive protein (CRP) and interleukin-6 (IL6) [10]. The etiology of inflammation in this case is multifaceted and involves patient-related causes, such as underlying disease, comorbidity, oxidative stress, infections, obesity, genetic or immunologic factors, or on the other hand, hemodialysis-related factors, mainly concerning the dialysis membrane biocompatibility and dialysate quality [11].

Secondary hyperparathyroidism is known to be another cause of morbidity and mortality in patients with end stage renal disease [12]. Growing evidence implies that higher levels of parathyroid hormone may be accompanied by low-grade inflammation but this association remains uncertain. Parathyroid hormone encourages interleukin-6 (IL-6) production by osteoclast and liver cells [13]. Other studies showed elevated levels of C-reactive protein (CRP), tumor necrosis factor-a and other inflammatory markers in hyperparathyroidism patients [14] [15].

The aim of the current study was to investigate serum levels of CXCL16 in patients undergoing hemodialysis and their correlation with other inflammatory markers such as highly sensitive C-reactive protein (CRP) and uremic toxins as intact parathyroid hormone (iPTH).

## Materials and Methods

The present study was performed on a selected group of 40 patients (22 males) with diagnosed end stage renal disease from those attending the hemodialysis unit of Cairo University hospitals in the period from October to December 2018. Their mean age was 47.6 ± 13.4. All underwent dialysis three times weekly during a period of four hours using synthetic membranes (Fresenius polysulfone UF 4.0; Fresenius Medical Care, Hamburg, Germany). All patients fulfilled the criteria of adequate dialysis (Kt/V≥ 1.2 according to the Daugirdas II formula). All patients had either hypertension or diabetes mellitus or both. The study included 10 patients (25%) with hypertension, 14 patients (35%) with diabetes mellitus and 16 patients (40%) with both hypertension and diabetes. Patients with acute infections, cancer, hepatic disease, heart failure and myocardial infarction were excluded from the study. Blood samples were withdrawn from the arteriovenous fistula of hemodialyzed patients before starting the dialysis session. Informed consent was obtained from all participants in the study. This study followed the ethical standards of the national research committee given in the Declaration of Helsinki 1964, as revised in 2013. In addition, this study followed the ethical standards of the National Center for Radiation Research and Technology (NCRRT) Ethical Committee (Cairo) and this work was given the approval code 8H/19. The authors affirm that they have no conflict of interest. The control group comprised another group of 40 age and gender matched apparently healthy subjects (24 males). Their mean age was 45.4 ± 15.6. They attended their routine visits at the preventive health service clinic on the same day of patients' dialysis appointments. They were considered as healthy volunteers with normal complete blood count, fasting blood glucose, liver and kidney functions.

A detailed medical anamnesis was obtained from patients and controls, and a careful clinical examination was completed for them.

Blood samples were collected from all participants in suitable vacutainers. Sera and plasma were separated as the preferred schedule and kept at -20 °C till assay time. Fasting blood sugar, urea, creatinine, calcium and inorganic phosphorous were measured using routine methods on Hitachi 971 instrument (Roche Diagnostics GmbH, D, 68298 Mannheim). glycosylated hemoglobin (HbA1c) was determined by quantitative chromatographic spectrophotometric determination using a kit provided by Biosystem reagents and instruments, Barcelona (Spain) [16]. Highly sensitive C-reactive protein (CRP) was assayed by nephelometry using BN-Prospect system (Dade Behring, GmbH, Marburg, Germany) [17]. Intact PTH (iPTH) levels were determined by intact PTH assay using chemiluminescent microparticle immunoassay on the ARCHITECT I system provided by ABBOTT Diagnostics Division, Biokit S.A. 08186 Barcelona, Spain [18]. Serum CXCL16 levels were measured using a suitable ELISA kit purchased from Wuhan EIAAB Scientific Co., China according to the manufacturer's instructions.

### Statistical Analysis

All data in this work was expressed as mean ± standard deviation. Correlations between Serum CXCL16, CRP, PTH as well as other variables were evaluated by Pearson's Correlation test. Comparisons between the means of two groups were evaluated using Student's two tailed t-test. All statistical analysis was carried out using Statplus version 2 for Macintosh statistical software.

## Results

**Figure 1:**
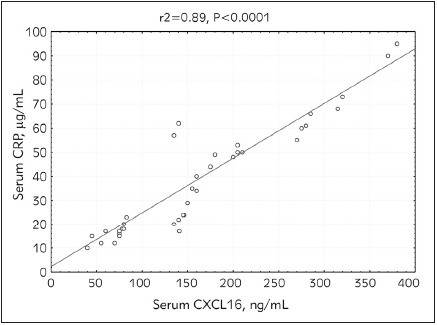
Correlation between CXC chemokine ligand - 16 (CXCL-16) and C-reactive protein (CRP) in end stage renal disease (ESRD) patients on hemodialysis.

**Figure 2:**
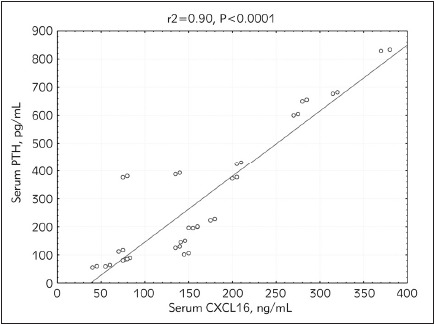
Correlation between CXC chemokine ligand 16 (CXCL16) and intact parathyroid hormone (iPTH) in end stage renal disease (ESRD) patients on hemodialysis.

**Figure 3:**
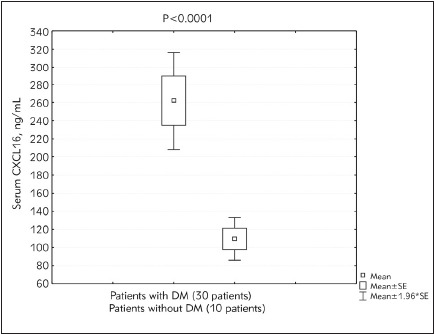
Serum CXC chemokine ligand 16 (CXCL16) in end stage renal disease (ESRD) patients on hemo dialysis with and without diabetes mellitus (DM).

**Figure 4:**
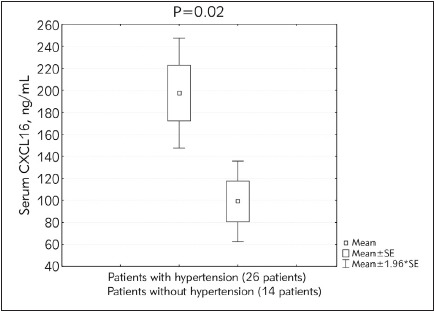
Serum CXC chemokine ligand 16 (CXCL16) in end stage renal disease (ESRD) patients on hemo dialysis with and without hypertension.

The current study included a total of 40 patients on hemodialysis (30 patients had diabetes mellitus while 26 patients were hypertensive). Serum CXCL16, CRP, PTH, FBS, urea, and creatinine levels were statistically significantly higher in patients participating in the study compared to the controls (p<0.0001). Glycosylated hemoglobin percentage (HbA1c) and phosphorus levels were also found to be significantly higher in hemodialysis patients compared to the controls (p<0.0004). However, no statistically significant differences were observed in the cases compared to the controls as regards calcium levels. These findings are presented in Table 1 below. Serum CXCL16 levels correlated positively with CRP (r=0.956, p<0.00001). Also, a highly significant positive correlation was detected between iPTH and CXCL16 (r =-0.403, p<0.001) (Figure 1 and Figure 2). Diabetic and hypertensive hemodialysis patients demonstrated highly significant CXCL16 levels compared to those who were non-diabetic or non-hypertensive respectively (Figure 3 and Figure 4)

**Table 1:** Basic clinical and biochemical data for the patients vs. controls. N/A= Not Applicable. P<0.05 = Significant P<0.01 = Highly Significant

Parameter	Controls (n=40)	Patients (n=40)	P-value
Age (years)	45.4 ± 15.60	47.60 ± 13.40	1.50
Sex ratio (Male/Female)	24/16	22/18	N/A
CXC Chemokine Ligand-16 (μg/mL)	46.75 ± 7.78	162.95 ± 89.7	<0.0001
Parathormone (pg/mL)	39.55 ± 19.54	293.35 ± 234.1	<0.0001
C-reactive Protein (mg/mL)	3.80 ± 3.14	39.10 ± 23.69	<0.0001
Fasting glucose (mmol/L)	4.93 ± 0.38	8.60 ± 1.96	<0.0001
Glycosylated hemoglobin (%)	5.01 ± 0.47	5.93 ± 0.94	<0.0004
Creatinine (μmol/L)	90.16 ± 22.98	617.9 ± 155.58	<0.0001
Urea (mmol/L)	9.62 ± 2.22	42.40 ± 13.26	<0.0001
Calcium (mmol/L)	8.48 ± 0.47	8.79 ± 0.61	<0.08
Phosphorus (mmol/L)	3.46 ± 0.46	4.39 ± 1.26	<0.004

## Discussion

Patients on chronic hemodialysis are at a greater risk of morbidity and mortality that can not only be explained by traditional risk factors of atherosclerosis like diabetes, hypertension, dyslipidemia but also the other factors like inflammation [19]. The causes of inflammation in hemodialysis patients are multifactorial. An inflammatory reaction may originate from several sites including graft, fistula, dialysis membrane and chronic infections [20].

In the current study, there were increased levels of serum CXCL16 and CRP observed in patients on hemodialysis compared to the controls demonstrating that dialysis treatment and uremia may cause hyperactivation of the immune system leading to an elevation of peripheral markers of immune activation mainly cytokines and chemokines. All alterations in innate immunity are understood to lead to an elevation of cytokines and inflammatory markers [21]. Cytokines are known to be the chief controllers of host response to infection and inflammation and play a part in arterial disease and mortality in hemodialysis patients [22]. Furthermore, proinflammatory cytokines stimulate the release and synthesis of acute phase proteins such as CRP from hepatocytes during inflammation [23].

Under normal baseline conditions, inflammation is unanimously regarded as a protective and physiological reaction to various harmful stimuli. Nonetheless, in chronic kidney disease (CKD), persistent systemic inflammation is accompanied by increased mortality due to cardiovascular disease (CVD) and infectious complications related to immune dysfunction [24]. C-reactive protein seems to be connected to endothelial dysfunction and foam cell formation [25]. Rising evidence has implied that there is an association between inflammation and cardiovascular (CV) risk. However, a precise underlying relationship cannot be identified.

Secondary hyperparathyroidism is one of the first disorders in mineral metabolism in patients on hemodialysis. The increase of parathormone (PTH) is caused by acidosis, resistance to calcitriol, decreased blood calcium due to the reduction of the active form of vitamin D [26]. Parathormone is considered a uremic toxin which may inflict damage in multiple organs. Recently Cheng et al. [27] have shown that PTH levels have an impact on the symptoms and quality of life in dialysis patients with secondary hyperparathyroidism. Parathyroidectomy in these patients has been shown to improve their quality of life. Accumulating evidence suggests that higher PTH levels may be associated with low grade inflammation. The reason and effect association between PTH and inflammation remains uncertain.

In the present study, serum iPTH levels were significantly higher in dialysis patients compared to the controls. Moreover, a highly significant positive correlation was found between PTH and the inflammatory chemokine CXCL16. Also, CXCL16 levels were positively associated with CRP. Similar correlations were obtained by Elewa et al. [28] and Nazari et al. [29].

Our results pointed to an association between iPTH and inflammation. CRP is considered to be the most important marker of inflammation related to CV complications. Cheng et al. [27] found a significant positive correlation between PTH and CRP. Hu et al. [30] demonstrated for the first time that inflammation accelerated lipid accumulation in renal tubules and that tubular damage occurred via the upregulation of CXCL16 pathways and significantly accelerated renal failure. Furthermore, there is experimental evidence that CXCL16 may cause glomerular injury adding a further potential explanation to the clinical association observed between CXCL16, markers of inflammation such as CRP and uremic toxins such as PTH [26]. The results verify that CXCL16 levels increase considerably in chronic renal disease and are in congruence with the results observed by Nazari et al. [29] who revealed that CXCL16 levels were 2.5 times higher in CKD subjects than healthy controls. Therefore, it is reasonable that chronic inflammation could have a close relationship to hemodialysis.

Unal et al. [31] documented that increased highly sensitive c-reactive protein (CRP) and PTH levels was associated with increased serum CXCL16 levels. This association was evidenced by multiple stepwise regression analysis of variables that were independently associated with serum levels of CXCL16 including decreased eGFR, diabetes mellitus, hypertension, increased CRP and PTH levels.

Results of the present study showed that hemodialysis patients with diabetes or hypertension had significantly higher CXCL16 levels compared to hemodialysis patients without diabetes or hypertension respectively. Comparable results were reported by Lin et al. [32] who found higher CXCL16 levels in Chinese patients who were diabetic with CKD than those without diabetes mellitus. This could explain the in creased incidence of coronary artery disease (CAD) in those patients and the close relationship between CAD and increased levels of CXCL16. Another explanation is that CXCL16 by itself could be a cause of insulin resistance. Even though CRP levels are generally increased in inflammatory diseases, it seems that serum CXCL16 may be a better biomarker than CRP in diabetic patients with CAD.

Lv et al. [33] found an increase in the levels of soluble CXCL16 in patients with metabolic syndrome and documented that CXCL16 was a proinflammatory factor that was associated with plaque formation. Also, Elewa et al. [28] documented higher CXCL16 levels in patients with prior CVD on top of CKD compared to those with CKD per se. Additionally, Nazari et al. [29] observed a similar significant difference between hemodialysis patients and hemodialysis patients with CVD. There are several limitations in this study. A larger sample size may be needed for more comprehensive investigations. Parameters of lipid and antioxidant status were overlooked and the study focused on the inflammatory markers and this is due to the fact that many studies stated that they are more important in hemodialysis patients than traditional risk factors. In addition, serum albumin, LDL and HDL cholesterol levels are known to decrease with inflammation.

## Conclusion

In conclusion, patients on hemodialysis are at a greater danger of suffering an inflammatory reaction against factors originating from grafts, fistulae and dialysis membranes. These reactions are linked to increased levels of inflammatory markers such as CRP and cytokines such as CXCL16. CXCL16 could be used as an inflammatory biomarker together with CRP in hemodialysis patients. Hyperparathyroidism could be a condition related to the inflammatory status in hemodialysis patients.

## Conflict of interest statement

The authors declare that they have no conflicts of interest in this work.
